# The burden and treatment of diabetes in France

**DOI:** 10.1186/1744-8603-10-6

**Published:** 2014-02-20

**Authors:** Karine Chevreul, Karen Berg Brigham, Clara Bouché

**Affiliations:** 1 URC Eco Ile-de-France (AP-HP), Hôtel Dieu – Galerie B1 – 3ème étage, 1 Place du Parvis Notre Dame, 75004 Paris, France; 2 LIC EA 4393, University Paris-Est Créteil (UPEC), 61 avenue du Général de Gaulle, 94010 Créteil Cedex, France; 3 Endocrinology Department, Saint Louis Hospital (AP-HP), 1, avenue Claude-Vellefaux, 75010 Paris, France

**Keywords:** France, Diabetes, Complications, Cost, Chronic disease management

## Abstract

**Background:**

The objective of this review was to describe and situate the burden and treatment of diabetes within the broader context of the French health care system.

**Methods:**

Literature review on the burden, treatment and outcomes of diabetes in France, complemented by personal communication with with diabetes experts in the Paris public hospital system.

**Results:**

Prevalence of diabetes in the French population is estimated at 6%. Diabetes has the highest prevalence among all chronic conditions covered 100% by France’s statutory health insurance (SHI), and the number of covered patients has doubled in the past 10 years. In 2010, the SHI cost for pharmacologically-treated diabetes patients amounted to €17.7 billion, including an estimated €2.5 billion directly related to diabetes treatment and prevention and €4.2 billion for treatment of diabetes-related complications. In 2007, the average annual SHI cost was €6 930 for patients with type 1 diabetes and €4 890 for patients with type 2 diabetes. Complications are associated with significantly increased costs. Diabetes is a leading cause of adult blindness, amputation and dialysis in France, which also has one of the highest rates of end-stage renal disease in Europe. Cardiovascular disease is the leading cause of death among people with diabetes. Historically, the French health care system has been more oriented to curative acute care rather than preventive medicine and management of long-term chronic diseases. More recently, the government has focused on primary prevention as part of its national nutrition and health program, with the goal of reducing overweight and obesity in adults and children. It has also recognized the critical role of the patient in managing chronic diseases such as diabetes and has put into place a free patient support program called “sophia”. Additional initiatives focus on therapeutic patient education (TPE) and the development of personalized patient pathways.

**Conclusions:**

While France has been successful in protecting patients from the financial consequences of diabetes through its SHI coverage, improvements are necessary in the areas of prevention, monitoring and reducing the incidence of complications. Systemic changes must be made to improve the coordination and delivery of chronic care.

## Background

France has a social security type system of public health insurance with almost universal coverage [1]. Historically, individuals were covered based on employment; however, coverage changed to a citizenship basis in 2000 when the Universal Health Coverage Act (CMU) offered basic health insurance coverage to every resident of France regardless of employment status and medical assistance (*Aide médicale de l’Etat*; AME) for foreigners without resident status who have lived in France for more than three months.

The French health benefit basket is considered generous, although health goods and procedures are not 100% covered except for people with certain chronic conditions. Diabetes is one of 30 chronic diseases covered 100% by statutory health insurance (SHI) pursuant to the ALD scheme (*affections de longue durée*). For those not covered under ALD, a share of the official health care tariff is paid by the patient and varies depending on the category of goods and care.

Patients have access to both public and private hospitals, and outpatient care is generally provided by self-employed health professionals working in private practice. The SHI directly pays hospitals on a DRG basis, and the hospitals in turn bill the patient a lump sum per diem for hospital catering and the 20% co-payments when applicable. However, the latter is covered by voluntary complementary insurance for 94% of the population [2].

In the outpatient sector, services are covered if they are included in one of the SHI positive lists of reimbursable services and goods. In order to be eligible for reimbursement by the SHI, pharmaceutical products and medical devices must be prescribed by health care professionals (doctors, dentists and midwives). Doctors and other health professionals are usually paid on a fee-for service-basis by patients who then file claims for reimbursement. In the outpatient sector the share of the official tariff covered by the SHI ranges from 70% for health care provided by doctors and dentists to 60% for paramedical professionals and laboratory tests. Most drugs are covered at a rate of 65%, but this varies from 100% for non-substitutable or expensive drugs to 15% for drugs considered “convenience medications”. Certain medications are not covered by the SHI because their therapeutic value has been judged to be insufficient.

Recent reforms have aimed at improving efficiency and coordination of care, including a gatekeeping approach with patient-designated “preferred doctors”, pay for performance incentives and promotion of patient pathways for chronic diseases.

The objective of this article is to describe and situate the burden and treatment of diabetes within the broader context of the French health care system.

## Methods

Our study was based on secondary data analysis complemented by expert consultation. We undertook a review of the peer-reviewed and grey literature, including policy documents and governmental reports, as well as government statistics. The literature review was conducted in December 2010. We searched PubMed in both French and English, using the following key words: ((diabetes[Title]) AND France[Title/Abstract]) for the years 2000–2010. We also searched non-indexed peer-reviewed publications in France to ensure that we did not omit any important sources. Studies presenting national data on prevalence, incidence, mortality, screening, treatment, outcomes, costs and complications in the French population were included. Data and reports from governmental entities and professional societes were also reviewed. This evidence was complemented and confirmed by primary data obtained through personnal communication with French diabetes experts from the Assistance Publique-Hopitaux de Paris (AP-HP).

## Results

The search strategy yielded 213 articles, of which 184 were excluded because they did not meet the inclusion criteria or they were superceded by more recent or more complete national data. Thus, 29 peer-reviewed papers were included in our review (Table 1).

**Table 1:** Literature review results

**Aspect of diabetes management**	**Number of references retained**	**References**
Prevalence, incidence and mortality	4	[5,7,49,50]
Costs	6	[39,43,45-48]
Complications	13	[30-34,36-38,44,51,54-56]
Screening, treatment and outcomes	6	[20,23,25,26,42,53]

### Incidence

In the absence of an ongoing cohort or registry in France, the incidence of diabetes (new cases per year) is difficult to estimate. However, data from the SHI funds provide a precise picture of the patients currently treated for diabetes. A type 1 diabetes registry was maintained from 1988 until 1997 and found an incidence rate of 9.6 per 100 000 inhabitants in 1997 [3]. Type 1 incidence has doubled in 30 years for the 0–15 age group and doubled in 15 years for the 0–5 age cohort. Incidence of combined type 1 and type 2 diabetes has been estimated based upon new admissions to the ALD program. The incidence rate of ALD admissions for diabetes reached 289 per 100 000 inhabitants in 2006, which corresponds to approximately 178 000 new cases [4].

### Prevalence

Prevalence of diabetes in the French population is estimated at 6%, including patients treated with oral antidiabetic medications and/or insulin (4.4%), [5] patients treated with diet alone (0.6%) [6] and individuals with undiagnosed diabetes (1%) [7]. Type 2 diabetes accounts for the vast majority of cases (92%) [5]. Since 2010, diabetes has had the highest prevalence among all ALD conditions, and the number of patients covered has doubled in the past 10 years [8].

### Demographics

The French population with diabetes is older (average age 65), majority male (54%), with a significant percentage of immigrants (23% born outside of France, compared to 8% of the general population) [5].

### Policies

There is no current national plan for diabetes in France, although such plans exist for other diseases, including Alzheimer’s disease (2008–2012), [9] cancer (2009–2013) [10] and HIV/AIDS (2010–2014) [11]. From 2002–2005 a national program for type 2 diabetes focused on prevention, screening, the quality and organization of treatment, epidemiology and patient education and led to ongoing initiatives [12]. In addition, a national plan to improve the quality of life of persons with chronic diseases (2007–2011) has emphasized therapeutic education and improved epidemiological data collection as priorities [13].

The ENTRED studies (*Echantillon national representative des diabétiques*) were carried out by the National Institute for Public Health Surveillance (*Institut de Veille Sanitaire*; InVS) from 2001–2003 and 2007–2010 and constitute one of the most important diabetes initiatives of the past decade. Based upon random samplings of adult SHI beneficiaries who had received at least three reimbursements for oral antidiabetic medications or insulin over a 12-month period, the ENTRED studies supplemented these data with hospitalization records, telephone interviews of physicians, as well as postal surveys of diabetic patients and their treating physicians. Although the ENTRED studies were limited to patients who were treated pharmaceutically for diabetes, they have yielded important insights, particularly regarding the evolution of diabetes and related complications in France.

Based upon the results of the ENTRED 2001 study, the government has focused attention on diabetes complications. A 2004 public health law set two goals with respect to diabetes: ensuring that at least 80% of diabetic patients receive the monitoring examinations recommended by clinical guidelines and reducing the frequency and severity of diabetic complications, particularly cardiovascular complications [14].

Finally, the government has focused on primary prevention as part of its national nutrition and health program (*Programme national nutrition et santé*; PNNS), with the goal of reducing overweight and obesity in adults and children [15]. In 2009, 31.9% of French adults were overweight (BMI 26–30 kg/m^2^) and 14.5% were obese (BMI ≥30) [6]. Overweight increases the risk of developing type 2 diabetes three times, and obesity increases that risk seven times. The National health authority (*Haute autorité de santé*; HAS) has updated its recommendations for treatment of obesity in adults as well as in children and adolescents. In addition to addressing overweight and obesity, the expert committee for PNNS 2011–2015 has proposed more aggressive screening of persons with pre-diabetes, in particular those with glucose intolerance [16]. Currently, oral glucose testing (fasting or non fasting) in France is generally limited to pregnant women in order to diagnose gestational diabetes.

### Health policy in relation to disease management

The French government has recognized the critical role of the patient in managing chronic diseases such as diabetes. A patient support program, “sophia”, was developed by France’s largest health insurance fund to provide free information and educational tools to diabetes patients covered by the ALD program [17]. The project, which began as a pilot in 2008 and was expanded nationwide in early 2013, has to date provided services to 226 000 patients (12.5% of the eligible population) [18]. Participation is voluntary, and the services offered include telephone advice by specially-trained nurses as well as Internet-based support to ensure regular contacts with patients.

In addition to the “sophia” initiative, the government has defined therapeutic patient education (TPE) as a national priority as part of the major health system reforms passed in 2009 [19]. While such educational programs have been offered by a number of diabetes provider networks in France, the programming, financing and participation are heterogeneous. Only 2.5% of treated diabetic patients reported that they were part of a diabetes network [20]. The law sets standards for TPE programs, which are now subject to authorization by the regional health agencies. Pluridisciplinary teams (which must include a physician) first analyze a patient’s needs in order to set personalized educational objectives. The education itself can take place one-on-one or in a group setting or both and must be evaluated to ensure that the objectives are met. However, the financing mechanism for this initiative has not yet been defined.

The National health authority is developing new tools for health professionals and patients with chronic diseases to facilitate the design of personalized patient pathways [21]. To date, guides and associated tools for four chronic diseases have been published and another four are being prepared, although diabetes is not among them.

### Diabetes treatment

In France, screening for type 2 diabetes is done based on clinical signs (e.g., polyuria/polydypsia) as well as on an opportunistic basis, targeted at individuals age >45 with at least one of the following risk factors: body mass index (BMI) ≥28 kg/m^2^; blood pressure ≥140/≥90 mmHg; HDL cholesterol ≤0.35 g/L and/or triglycerides ≥2 g/L and/or treated high cholesterol; family history; gestational diabetes or children with birth weight over 4 kg; temporarily induced diabetes [22]. Screening is done via a fasting serum glucose test. The overall opportunistic screening rate over two years was 48.6% overall and increased with age [23]. Among those over age 45, the screening rate increased to 71.2% and was higher among women than men. Populations that may be missed by this targeted screening approach include those who do not use medical services and at-risk populations, such as homeless people.

Treating diabetes is complicated because of the need to normalize the glycemic level and to address any cardiovascular risk factors or existing complications, while taking into consideration the individual needs and characteristics of the patient. Clinical treatment recommendations for type 2 diabetes have existed in France since 1999, with the most recent revision issued in January 2013 [24]. The general philosophy behind the guidelines is that treatment should be individualized and thus evolve over time based on regular re-evaluation of all aspects of treatment: life style, therapeutic education and medication. Physicians appear to have a good awareness of the recommendations, [25] but they do not strictly apply them in managing their patients with type 2 diabetes [26].

The type and intensity of treatment is based on the patient’s medical history and a range of outcome measures: laboratory tests for glycemic control, blood lipids, creatinine and urinary proteins, and clinical screening for ophthalmological, cardiac and podiatric complications. Thus regular monitoring is essential to ensure appropriate and timely treatment of diabetes and its complications.

#### Physicians

Most people with type 2 diabetes are treated by general practitioners (GPs), very few of whom have specialized training in diabetology/endocrinology or nutrition [20,27], There is no recognized specialty of diabetology in France, although there are endocrinologists who limit their practices to diabetes. In 2007, only 10% of patients with diabetes (generally patients with type 1 diabetes and some patients with type 2 diabetes treated with insulin) had a consultation with an endocrinologist [20]. There are only 1.25 endocrinologists in the ambulatory sector per 100 000 inhabitants, with large geographic disparities [28]. The majority of endocrinologists (64.74%) practice in “sector 2”, meaning that they may charge fees in excess of the officially set tariffs, which are not covered by the ALD program. By contrast, 92% of GPs practice in “sector 1” and thus accept the statutory tariffs.

GPs receive €40 per ALD patient per year to offset the time involved in coordinating with specialists. In addition, pay-for-performance (P4P) incentives have been implemented to promote quality and efficiency in primary care. They do not change the basis of fee-for-service payment but offer additional remuneration to GPs meeting defined objectives. Among the 29 indicators, eight specifically target diabetic patients, focusing on HbA1c testing and results, LDL cholesterol testing results, biennial eye examinations and treatment with antihypertensives/statins and anticoagulant/antiplatelet medications.

#### Paramedical professionals

Access to paramedical professionals appears to be limited. Only 20% of type 2 diabetic patients reported having a consultation with a dietitian in 2007 [20]. Such visits were generally related to insulin treatment and thus late in the evolution of type 2 diabetes. Dietitian visits are not covered by SHI, and consultations with podiatrists have only recently become covered for patients with grade 2–3 lesions [29]. Patients reported low rates of consultations with podiatrists/chiropodists (23%) and nurses (26%). There are no nurse practitioners in France.

#### Screening and treatment of complications

With respect to diabetic eye disease, there is a lack of qualified professionals for ophthalmological screening, in part because there are no optometrists in France [30]. In 2009, there were only 5 567 ophthalmologists, which equates to one for every 520 persons with diabetes [27]. The growing number of people with diabetes and the decreasing number of ophthalmologists able to perform fundoscopic examinations has been cited as a contributing factor to access problems [31]. To address this challenge, French regions have deployed various innovative methods to screen for diabetic retinopathy, ranging from the Ophdiat telemedical network in the Ile-de-France [32] to Bourgogne’s mobile screening units [33]. Nonetheless, regional variations in access to ophthalmological screening remain [34].

Screening for foot disease does not require referral to a specialist. Indeed, the French diabetes society recommends a clinical examination of the feet of diabetic patients at each visit, even in the absence of symptoms [35]. However, an ENTRED study found that only 20% of patients questioned said that they had received a screening with monofilament [36].

The control of vascular risk factors improved between 2001 and 2007, likely due to the intensification of pharmacological treatment with antihypertensive and cholesterol-lowering medications [37]. The majority of type 2 diabetic patients were treated with antihypertensive drugs (75%) and cholesterol-lowering drugs (59%) [20]. Nonetheless, the frequency of coronary complications has not diminished since 2001, and only 14% of patients with type 2 diabetes have blood pressure below the recommended level of 130/80 mm/Hg.

Renal complications are likely underestimated and thus under-screened due to the fact that patients tend to remain asymptomatic for a long time. The fact that one in three diabetic patients suffering renal failure began dialysis under emergency circumstances has been cited as evidence of late referral to nephrologists [38].

### Finance and organization of health care delivery

Diabetic patients are eligible to apply for ALD coverage from the time they are diagnosed. ALD 8 includes diabetes types 1 and 2, and 84% of diabetic patients are covered under this program [39]. The list of procedures and services covered under ALD is comprehensive [40] and includes virtually all medications with few exceptions. However, services not otherwise covered by SHI are not eligible for coverage under ALD unless they are provided in a hospital or network setting.

Since the passage of the 2009 Hospital, Patients, Health and Territories Act, [19] 26 regional health agencies have been charged with identifying health needs in light of the care capacity of the region and defining strategic priorities. The populations targeted by these regional strategic plans (*plans stragégiques regionaux de santé*; PSRS) include persons with chronic diseases, with a particular focus on prevention, TPE and patient pathways.

### Costs

In 2010, SHI cost for pharmacologically-treated diabetes patients amounted to €17.7 billion (Table 2). This amount includes the cost for all care of the person with diabetes, whether for diabetes or another illness. An estimated €2.5 billion was directly related to treatment of diabetes and prevention, while €4.2 billion was for treatment of diabetes-related complications. An additional €3.5 billion was attributable to comorbidities that are more frequent among diabetic patients, particularly the most disadvantaged, such as cancer and obesity [41].

**Table 2:** SHI annual expenditure for pharmacologically-treated patients with diabetes, 2010

		**Total cost (€ billions)**
**Screening**		**0.03**
**Treatment/prevention of complications**		**2.5**
Glycemic control	1.7	
Screening of complications	0.1	
Prevention of cardiovascular complications	0.8	
**Treatment of diabetes-related complications**		**4.2**
Cardiovascular	0.8	
Renal insufficiency	0.7	
Neuropathy	0.3	
Vision loss	0.1	
Other complications	0.3	
Health expenditure indirectly related to diabetes*		**3.6**
Health expenditure unrelated to diabetes**		**7.4**
**Total health expenditure for patients with treated diabetes**		**17.7**

*Expenditure related to pathologies frequently found in patients with diabetes (e.g., certain cancers, obesity).

**Expenditure for patients with diabetes a priori unrelated to diabetes, including the share of the cost of complications not attribuk to diabetes, as well as other health expenditures for these patients that are not diabetes-related.

Source: CNAMTS [41].

In 2007, the average annual SHI cost was €6 930 for type 1 diabetic patients and €4 890 for type 2 patients. For insulin-treated type 2 diabetic patients, the annual SHI cost increased to €10 400. SHI cost per person treated for diabetes increased 30% between 2001 and 2007, an average annual increase of 4.4%. The total SHI cost for patients treated for diabetes increased 80% in constant euros between 2001 and 2007 due to the increase in prevalence of diabetes (+38% over seven years), as well as serious and costly complications and hospitalizations [39].

Hospital charges accounted for 37% of SHI cost, and 31% of patients treated for diabetes were hospitalized during the one-year study period [39]. The hospital expenditures for diabetic patients are likely even higher because diabetes is not necessarily included as a secondary diagnosis in France’s hospital data collection system (*Programme de médicalisation de systèmes d’information*; PMSI), which also does not reveal patients’ ALD status. One study found that diabetes diagnosis is not mentioned in 51.3% of hospitalizations or for 29.3% of patients [42]. Moreover, hospitalizations for cataracts and dialysis are not considered diabetes-related hospitalizations (the annual reimbursement for a diabetic end stage renal disease (ESRD) patient is estimated at €65 000) [43]. Diabetes has a significant impact on hospitalization costs in part because it increases the length of stay. For example, the average length of stay of diabetes patients following cardiovascular events was longer (stroke: +2.5 days, myocardial infarction: +1.5 days, unstable angina: +1.3 days, revascularization +2.8 days) and thus more costly (non-fatal stroke: +23.9%, non-fatal myocardial infarction: +10.4%, unstable angina: +6.1%, coronary revascularization: 9.1%) than for non-diabetic patients [44].

Pharmaceutical expenses comprised 27% of total cost, with cardiovascular drugs (€1.25 billion) accounting for a significantly higher share of the cost than oral antidiabetic medications and insulin (€770 million) [39].

Complications among people with type 2 diabetes are associated with significantly increased costs [45]. Four complications account for nearly 9% of medical costs for type 2 diabetes: recent myocardial infarction; stroke resulting in invalidity; chronic renal disease; and peripheral arterial disease [46]. Macrovascular complications (myocardial infarction, heart attack, angina, coronary revascularization, stroke) result in medical costs that are 1.7 times higher; costs for microvascular complications (ophthalmological laser treatment, blindness in one eye, amputation, existing or treated diabetic foot) are 1.1 times higher in persons with type 2 diabetes; and end stage renal disease (requiring dialysis and/or transplant) multiply the costs by 6.7 times. Application of treatment guidelines has been shown to result in cost savings [47].

The ENTRED cost data do not include the costs for diabetic patients not pharmacologically treated, nor do they include patients’ out-of-pocket expenses or contributions for complementary insurance. Moreover, the costs related to diabetes are not only medical and include loss of productivity and support payments. For example, disability pensions for 3.6% of persons with diabetes under the ALD regime average €7 060 per year, and daily allowances averaging €2 661 per year are paid to 8.5% of persons with diabetes under the ALD regime [48].

### Outcomes

#### Mortality

Diabetes was mentioned among the diseases contributing to death on 6.1% of death certificates in 2006, with 2.2% noting diabetes as the primary cause of death [4]. However, diabetes mortality data have been found to be under-reported by 20%, [49] which would increase the rate to 7.3%.

#### Prevalence of complications

Uncontrolled blood sugar can lead to microvascular complications (eye, nerve and kidney damage) and macrovascular complications (heart disease, stroke, peripheral arterial disease of the lower extremities, gangrene, abdominal aortic aneurysm). Given the difficulties in estimating the diabetes prevalence rate, it is not surprising that the data regarding diabetic complications are even more scarce. However, the significant consequences of these complications underscore the importance of better understanding their burden. Diabetes is a leading cause of adult blindness, [31] amputation [50] and dialysis [51] in France, and cardiovascular disease is the leading cause of death among people with diabetes [49].

##### Eye disease

The prevalence of diabetic retinopathy, based upon physician reports, has been estimated at 10% of treated diabetic patients [36,52]. However, it is likely that only the most serious cases were reported. Indeed, 16.6% of persons with diabetes stated that they had received an ophthalmologic laser treatment, [37] which is performed at the more severe stages of the disease. Thus, the prevalence of this complication can be assumed to be higher when patients not requiring laser treatment are included. Population-based studies in other countries have found the prevalence of diabetic retinopathy to be nearly three times higher (28.7%), [31] which may indicate that the disease is under-reported or insufficiently recognized by doctors in France.

##### Foot disease

Data on the prevalence of foot disease among diabetic patients are inconsistent, ranging from 15.3% [53] to 2.1% [52]. Among patients taking diabetes drugs, 9.9% reported having chronic foot ulcers [37]. Amputations, which are preceded by foot ulcers in 85% of cases, [54] affected 1.5% of diabetic patients in 2007 and accounted for 40-42% of the surgical hospitalizations of diabetic patients.

##### Cardiovascular disease

Coronary complications are the most frequent complication among drug-treated diabetic patients in France, and yet the prevalence data are far from clear. The most recent data regarding cardiovascular disease (CVD) among type 2 diabetic patients are based upon patient and physician surveys, with patients declaring complications more frequently than physicians [37]. Angina or myocardial infarction was reported by 16.7% of patients with type 2 diabetes, while 13.9% said that they had undergone coronary revascularization. Treating physicians reported heart failure (6.3%) and stroke (5%) among their diabetic patients.

##### Renal disease

Diabetes underlies 37% of new cases of ESRD, which requires dialysis and/or kidney transplant and affected 7 891 diabetic patients in France in 2006 [55]. With an incidence rate of 126 per 100 000 persons with diabetes, France has one of the highest rates of ESRD in Europe [38]. Diabetic patients make up nearly a quarter (23.6%) of patients receiving dialysis.

## Discussion

Like many countries, France has struggled to adapt a health system designed to treat acute conditions to the growing need for coordinated chronic care. In a 2008 survey of eight OECD countries by the Commonwealth Fund, France ranked lowest for chronic care management [56]. With respect to diabetes specifically, France had the lowest share of diabetic patients receiving all four recommended monitoring tests (HbA1c, cholesterol, feet and eye examinations). The ENTRED 2007 study revealed that only 2% of treated diabetic patients received all of the recommended annual examinations and laboratory testing [20]. This may explain the relatively high prevalence of complications such as ESRD in France.

The acute care model is also not adapted to a disease for which primary prevention is the essential element in slowing its progression. Given that the health determinants implicated in any prevention program are nutrition and physical exercise, policies must extend beyond health into the social sphere and also target the most vulnerable populations. However, the existing structures are fragmented, local and often not evaluated, and resources for implementation of programs shown to be effective are limited [8].

The need to improve the organization and coordination of diabetes care is widely acknowledged. Indeed, initiatives dating back more than 20 years have sought to address this gap, with a particular focus on the creation of diabetes networks. However, in addition to a low participation rate, the impact of such networks may be limited by the fact that they are external to GP practice, which is particularly problematic given the absence of electronic records to facilitate exchanges of information between the networks and GPs. Likewise, the effectiveness of the “sophia” program has not yet been demonstrated in terms of clinical outcomes [8]. This may be attributable in part to the voluntary, “opt in” nature of the program, which has resulted in a selection bias favoring less sick, more motivated patients. Nonetheless, an evaluation of the program found improved monitoring in accordance with the recommendations among the “sophia” patients as well as slower growth in hospital expenditures.

The recent focus on therapeutic patient education is an important step in addressing an issue that both patients and doctors have identified as essential to managing diabetes. It implicates a coordinated, pluridisciplinary approach that is currently missing in a system dominated by independent physicians reimbursed on a fee-for-service basis and in which there is no (or limited) reimbursement of paramedical professionals, such as dietitians and podiatrists. While TPE has been provided on a limited basis by diabetes networks, the 2009 law sets standards for such programs, which now must be authorized by the regional health authorities. However, significant details – most importantly, the financing of TPE programs – remain unresolved at this time.

Even if TPE programs for diabetic patients are extended, other issues, such as insufficient numbers of specialists (e.g., ophthalmologists) and regional disparities in access to certain services, will have to be addressed. This is one aspect of the broader problem of inadequate monitoring of outcomes essential to determining appropriate treatment strategies and early identification of complications. Innovations such as telemedicine may aid in addressing the problem of limited specialists. However, the fact that only 56% of patients are treated in accordance with the recommended objectives for glycemic control [20] suggests the need for improved support for clinical decision making, via initial and continuous medical education and reinforced by information systems and adapted payment schemes.

In addition, epidemiological surveillance is essential in order to understand the evolution of this growing health crisis and to develop effective measures to address it. While the ENTRED studies provided important evidence regarding diabetes in France over the past decade, the rising incidence points to the need for ongoing surveillance and improved data. At the moment, future plans for diabetes surveillance in France have not been announced.

## Conclusions

In its report evaluating the treatment of diabetes in France, the Inspector of Health and Social Affairs summarized the current situation: “The system is passive when it should be proactive with the chronically ill, prescriptive when it should support the patient in managing his illness, compartmentalized among health professions when it should be coordinated and multidisciplinary in its interventions” [8]. While the full range of curative treatments is available and accessible thanks to the French SHI coverage of all patients, prevention and monitoring of complications must be improved. Systemic changes in the coordination and delivery of diabetes care as well as improved epidemiological surveillance are necessary in order to better respond to the growing burden of diabetes in France.

## Abbreviations

ALD: Chronic disease coverage program; BMI: Body mass index; CMU: Universal health coverage act; CVD: Cardiovascular disease; ENTRED: Studies based on random sample of people pharmacologically treated for diabetes; ESRD: End-stage renal disease; GP: General practitioner; HAS: National health authority; InVS: National institute for public health surveillance; PMSI: Hospital data collection system; PNNS: National nutrition and health program; PSRS: Regional strategic health plan; SHI: Statutory health insurance; “sophia”: Chronic disease management program; TPE: Therapeutic patient education.

## Competing interests

The authors declare that they have no competing interests.

## Authors’ contributions

KC conceived the review and supervised all aspects of the research and manuscript preparation. KBB undertook the original literature search and CB provided expert input. KBB prepared the draft manuscript, which was reviewed by KC and CB, who provided additional material. All authors have read and approved the final manuscript.
